# Outdoor physical activity and self rated health in older adults living in two regions of the U.S.

**DOI:** 10.1186/1479-5868-9-89

**Published:** 2012-07-30

**Authors:** Jacqueline Kerr, James F Sallis, Brian E Saelens, Kelli L Cain, Terry L Conway, Lawrence D Frank, Abby C King

**Affiliations:** 1 Department of Family & Preventive Medicine, UCSD, San Diego, CA, USA; 2 Department of Pediatrics, Seattle Children’s Research Institute and University of Washington, Seattle, WA, USA; 3 Department of Psychology, San Diego State University, San Diego, USA; 4 School of Community and Regional Planning, University of British Columbia, Vancouver, Canada; 5 Department of Health Research & Policy and the Stanford Center for Research in Disease Prevention, Department of Medicine, School of Medicine, Stanford, CA, USA; 6 University of California, 9500 Gilman Drive, #0811, La Jolla, San Diego, CA, 92093-0811, USA

**Keywords:** Built environment, Accelerometers, Quality of life, Exercise

## Abstract

**Background:**

Older adults spend little time outdoors and many are physically inactive. The relationship between outdoor physical activity and self rated health has not been studied in older adults. This paper aimed to assess the relation of location of physical activity to self rated health and physical activity minutes. This was an observational study of ambulatory adults 66 years and older conducted in 2005–2008. Participants (N = 754) completed survey measures of physical activity location and self rated health, and wore an accelerometer to objectively assess physical activity. A mixed model linear regression procedure adjusted for neighborhood clustering effects. Differences in self rated health and physical activity minutes were compared across three physical activity settings (indoor only, outdoor only, both indoor and outdoor).

**Results:**

Minutes of moderate to vigorous physical activity were significantly greater in those who were physically active at least once a week outdoors compared with those who were physically active indoors only. Self rated health was significantly related to being physically active but did not vary by location of activity.

**Conclusions:**

Older adults who were physically active outdoors accumulated significantly more physical activity, but self-rated health was not significantly greater than those being physically active indoors.

## Introduction

Humans have an innate connection to nature known as ‘biophilia’ [1]. Exposure to nature has been shown to have restorative health effects, including recovery from surgery [2,3]. Studies have shown that going outdoors can have long term health benefits, particularly for older adults [4,5] who often have Vitamin D deficiency which is related to chronic conditions such as heart disease and bone health [6]. Vitamin D deficiency may be related to limited outdoor time in older adults, and outdoor activities may increase Vitamin D levels. For example, leisure time physical activity in a large US national sample was related to higher Vitamin D levels in older adults [7]. The occurrence of physical activity in outdoor locations may provide enhanced physical and mental benefits (e.g. reduced depression, increased bone health) as participants would experience both the benefits of exercise and Vitamin D [8,9].

Several studies documented positive effects of outdoor physical activity on mental health and well being in younger populations [10]. Outdoor exercisers may enjoy the activity more, performing it longer or more frequently, and benefit from social interaction afforded in outdoor locations [11]. The relation between physical activity location and health has not been studied specifically in older adults. Physical functioning limitations, fear of falling, and neighborhood design may prevent older adults from exercising outdoors [12-14].

This study of older adults investigated physical activity locations and their relation to objectively measured physical activity and self rated health. It was hypothesized that individuals exercising outdoors would exercise for longer periods and have greater self-rated health. This question is important because understanding activity patterns by location may help in the design of interventions to increase outdoor activity in older adults, which may result in greater health benefits, both from the longer exercise periods and exposure to healthful Vitamin D.

## Methods

The Senior Neighborhood Quality of Life Study was an observational study of ambulatory adults 66 years of age or older in 2 major US metropolitan regions selected to vary in neighborhood income and walkability conducted in 2005–2008. Study methods have been described elsewhere [15]. The study was approved by appropriate institutional review boards.

Participants (n = 896) completed two surveys and wore an accelerometer for 7 days. For these analyses, demographic data, self-rated health, and physical functioning were taken from survey one, physical activity location from survey two (conducted 6 months later). Five questions developed by investigators assessed physical activity location: a) indoors at home or apartment building, b) other indoor settings like recreation facilities, c) outdoors in a green or open space, d) outdoors in local streets or neighborhood, and e) outdoors outside of local neighborhood. Response options were: “more than once a week”, “once a week”, “less than once a week”, and “hardly ever”. The response categories were collapsed to those who were active in the location at least once a week versus less often, and four discrete groups were constructed: those who were *infrequently active* (i.e., less than once a week or hardly ever across all 5 questions), those who were physically active at least once a week *indoors only*, those who were physically active at least once a week *outdoors only*, and those who were physically active *both indoors and outdoors* at least once a week. These groups were chosen to control for the number of locations and isolate indoor and outdoor effects.

Self-rated health was measured by the question “In general, how would you say your health is, on a scale of 1–5, excellent to poor?” from the 12-item short-form health survey [16]. The scoring was reversed for these analyses so higher scores indicated better quality of life. Mobility impairment was assessed using the validated 11-item advanced lower-extremity subscale of the Late-Life Function and Disability Instrument [17], which assesses a broad range of functional capabilities requiring lower-body function (e.g., walking several blocks, going up and down 3 flights of stairs, getting up from the floor).

Ambulatory assessment of moderate-to-vigorous physical activity (MVPA) was accomplished using the extensively validated Actigraph accelerometer (Actigraph, LLC; Fort Walton Beach, FL, model 7164 or 71256) [18]. Participants were instructed to wear the device for 7 days. Data were collected in 1-minute epochs and cleaned and scored using MeterPlus version 4.0 software (Santech, Inc.; www.meterplussoftware.com). Scoring of MVPA was based on a commonly used cut-point (≥1952 counts/minute), [19] and derived as average minutes of MVPA per valid wearing day. A valid accelerometer hour was defined as having less than 45 consecutive minutes of ‘zero’ counts, and a valid day consisted of at least 8 valid hours.

### Analyses

A mixed model linear regression procedure was employed to adjust for neighborhood clustering effects. Minutes of MVPA per day and self-rated health were the dependent variables. The 4-category activity location was the independent variable. Post hoc pairwise comparisons were run to assess differences across the four groups. Covariates included age, gender, white/nonwhite ethnicity, education (college degree or not), neighborhood walkability and income (high vs. low; included because the study design created variability in these measures; walkability based on individual addresses was not included), and region of the US (Baltimore or Seattle). The model was run with and without physical functioning to assess its role as a mediator since previous studies have indicated low physical functioning may prevent outdoor activity, [12,13] and in our own previous analyses physical functioning moderated the relationship between the built environment and walking for transportation [20].

## Results

Of the 754 (84%) participants who completed the first and second survey and met the accelerometer wear time criteria, 23.6% were infrequently physically active (i.e. less than once per week), 25.8% were active at least once a week only indoors, 12.7% were physically active only outdoors, and 37.9% were active both indoors and outdoors at least once a week. The mean age of the sample was 75.2 years (SD 6.8), 55.2% were female, 48.9% were college educated, 71.1% were non-Hispanic white, 50.7% resided in low income neighborhoods, and 52.8% in high walkable neighborhoods. Half the sample lived in Seattle, WA (51.1%) and half in Baltimore, MD region.

**Figure 1:**
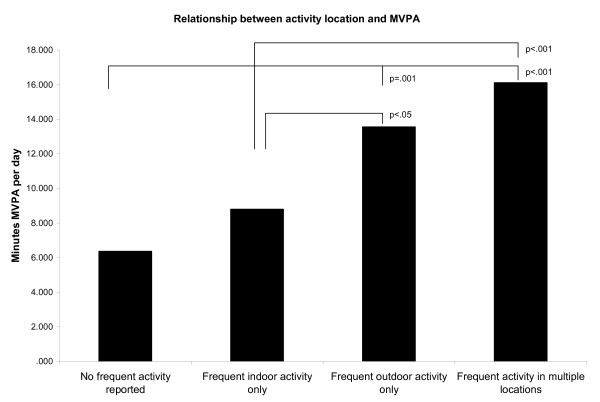
Relationship between physical activity location and minutes of MVPA from mixed methods adjusted model.

Figure 1 shows the relationship between reported physical activity location categories and daily minutes of MVPA from the adjusted mixed methods model (excluding physical functioning). The mixed methods regression analyses (excluding physical functioning) and post hoc tests indicated there was no significant difference in MVPA between older adults who reported *indoor only* physical activity versus *no* regular physical activity. Those who reported physical activity *outdoors only* did significantly more MVPA than those who reported *indoor only* or *no* regular physical activity. Those who reported physical activity in *indoor and outdoor* locations also did more MVPA than those who did physical activity *indoors only* or reported *no* regular activity. After adjusting for physical functioning, the *outdoor only* exercisers were no longer significantly more active than the *indoor only* exercisers. All other associations remained significant. This suggests that physical functioning may mediate the relation between MVPA and location of activity.

The mixed methods regression analysis of the relation between physical activity location and self rated health (excluding physical functioning) indicated that more frequent physical activity in all settings was associated with higher self rated health (p < .01). Those not frequently active reported the lowest self rated health (x̄ 2.76, SE .08), those in all active categories reported higher self reported health (indoor only: x̄ 3.04, SE .08, outdoor only: x̄ 3.24, SE .11, outdoor & indoor: x̄ 3.16, SE .07). *Outdoors only* exercisers reported the highest self-rated health, but this was not significantly different from other groups in post hoc analyses. The direction of the results and lack of significance remained the same when physical functioning was tested as a moderator.

## Discussion

Older adults who were physically active only in outdoor locations had at least half an hour per week more MVPA than those who were physically active indoors only. After adjusting for physical functioning, however, there was no longer a significant difference between indoor only and outdoor only groups. This pattern suggests that functional impairments can affect both the location and amount of physical activity older adults accumulate; i.e., individuals with impairment may not be able to exercise for as long and may not be able to or willing to access outdoor activity locations. Although those who were physically active outdoors-only reported the highest self-rated health, these differences did not reach statistical significance.

Limitations of this study included a cross-sectional design, no objective measure of where people were physically active, and no measure of air quality which may also affect overall health [21]. Additionally, the self-rated health measure consisted of only a single (albeit extensively used) item, and no assessments of the quality of the indoor or outdoor environments were included. Strengths included objective measurement of physical activity and the inclusion of survey items to assess multiple activity settings. Participants were recruited from 2 regions and resided in a range of high and low walkable neighborhoods, providing greater generalizability of results.

Future studies in older adults could be improved with both objective measures of physical activity and outdoor time, including accelerometers, [18] GPS devices, [22] and person-worn cameras which can capture images of exposure to green environments [23]. A study comparing indoor and outdoor physical activity with an inactive outdoor and indoor control condition would help to tease out the unique effects of physical activity in outdoor locations while controlling for social interactions (including dog walking) that may be one mechanism for the benefits of outdoor physical activity, particularly in older adults [11]. Person-worn pollution monitors [24] could also be helpful in assessing air quality in both outdoor and indoor environments—an environmental exposure of particular importance to older populations. Access to, and quality of, parks and indoor recreation facilities may moderate efforts to be active outdoors.

In conclusion, those who were physically active outdoors accumulated significantly more minutes of MVPA than those engaging in indoor-only physical activity. Self rated health was significantly higher in all those who were regularly physically active vs. not active but did not vary significantly by exercise location. Interventions that improve lower-extremity physical function may be required before older adults will be comfortable exercising outdoors. If increased health benefits are shown for outdoor activity, efforts could be made to provide safe and attractive outdoor locations for physical activity for older adults [25].

## Competing interests

The authors declare that they have no competing interests.

## Authors’ contributions

JK, JS, AK, BS, LF made substantial contributions to conception and design. JK, JS, AK, KC, TC made substantial contributions to acquisition of data. JK, JS, AK made substantial contributions to analysis and interpretation of data. JK, JS, AK, made substantial contributions to drafting the article. JK, JS, AK, BS, LF, KC, TC made substantial contributions to revising it critically for important intellectual content. JK, JS, AK, BS, LF, KC, TC gave final approval of the version to be published.
